# Factors associated with higher caregiver burden among informal caregivers of Parkinson’s disease: A systematic review

**DOI:** 10.1097/MD.0000000000041275

**Published:** 2025-01-24

**Authors:** Jinwen Wu, Mengnan Liu, Mingtai Chen, Jinyi Xue, Yuan Zou, Ziwen Deng, Shufei Zhao, Xue Yang

**Affiliations:** a Department of Cardiovascular Medicine, the Affiliated Traditional Chinese Medicine Hospital, Southwest Medical University, Luzhou, China; b Department of Cardiovascular Disease, Shenzhen Traditional Chinese Medicine Hospital, Shenzhen, Guangdong, China.

**Keywords:** burden, caregiver, factor, Parkinson’s disease, systematic review

## Abstract

**Background:**

Parkinson’s disease is a progressive neurodegenerative disease and the care burden in informal caregivers is huge. Summarizing factors associated with the informal caregivers burden can improve our understanding of providing proactive support to informal caregivers caring for patients with Parkinson’s disease (PwP) at risk, and provides evidence for clinical practice.

**Methods:**

PRISMA guidelines were followed in this systematic review. We searched 9 databases in May 2024 for studies reporting data on factors associated with the care burden of informal caregivers for PwP.

**Results:**

We identified 16456 records of which 28 met inclusion criteria. Informal caregivers from 12 countries were included. There were 38 factors shown to be linked to higher care burden. The evidence indicated that informal caregivers who were depressed, caring for PwP with lower quality of life, caring for PwP with lower ADL scores, or caring for PwP with depression reported high care burden.

**Conclusion:**

Targeted interventions addressing those modifiable factors should be developed and investigated to lighten the care burden of informal caregivers for PwP.

## 1. Introduction

Parkinson’s disease (PD) is a progressive neurodegenerative disease associated with the degeneration of dopaminergic neurons and the consequent decrease in the neurotransmitter dopamine, resulting in motor and non-motor changes.^[1]^ As the elderly population grows, the incidence of Parkinson’s disease is expected to double by 2030 in the top 5 western European and top 10 world population countries, including China.^[2]^ It is also reported that the number of patients with PD in China will rise from 1.99 million in 2005 to 5 million in 2030, which will approximately account for almost half of the global number of patients with PD.^[2]^ In the United States, the direct and indirect costs of Parkinson’s disease, including treatment, social security payments, and lost income due to inability to work, are estimated to be nearly $25 billion annually.^[3]^ As the aging of populations around the world progresses, Parkinson’s disease is of increasing concern as a common disease of old age.

When a person is diagnosed with Parkinson’s disease, the primary caregivers are their family members, all of whom are informal caregivers, including spouses, siblings, children and their relatives.^[4]^ Previous studies had reported that the majority of Parkinson’s disease informal caregivers are women, primarily the spouses of people with Parkinson’s disease.^[5]^ Informal caregivers of Parkinson’s disease are involved in numerous aspects of caregiving, including assisting with the diagnosis and treatment decisions on Parkinson’s disease, helping with activities of daily life, managing therapy-related adverse reactions, and dealing with negative emotions.^[6]^ Due to the individualized and progressively evolving manifestations of Parkinson’s disease, there is an escalating reliance on caregivers, which subsequently intensifies caregiver burden.^[7,8]^

The concept of caregiver burden, initially studied in the 1860s, is widely recognized as a primary indicator of adverse experiences among caregivers.^[9]^ Defined by George and Gwyther,^[10]^ caregiver burden encompasses the physical, psychological, social, and economic challenges faced by family members caring for older adults with disabilities. Poulshock and Deimling^[11]^ argued that caregiver burden arises from the psychological, physical, and social dimensions of the difficulties that caregivers face as a result of their caregiving activities. Caregivers are emotionally, physically and financially affected by their roles and are at risk of depression, chronic illnesses and other disease.^[12,13]^ Some of them may face to work changes and privately pay for medical care,^[14,15]^ which comes at a significant cost to the informal caregivers, further increasing the burden on caregivers.

In recent years, there has been a growing interest in the physical and mental health of informal caregivers of patients with Parkinson’s disease (PwP) and an increasing number of studies related to care burden,^[15–19]^ but there are still relatively few care burden interventions for caregiver for caring PwP and their effectiveness is uncertain. Therefore, this study aims to summarize and evaluate the evidence on the factors influencing caregiver burden in Parkinson’s disease based on previous studies, and to identify targets for interventions to reduce caregiver burden, with the hope of providing a reference basis for clinical research.

## 2. Materials and methods

### 2.1. Study registration

This systematic review followed the Preferred Reporting Items for Systematic Reviews and Meta-Analyses (PRISMA) guidelines.^[20]^ And the review protocol was registered on PROSPERO (CRD42024538779).

### 2.2. Inclusion and exclusion criteria

#### 2.2.1. Inclusion criteria

1. Participants: The inclusion population were PwP and their informal caregivers.
2. Intervention measures: The studies that included any factor causing caregiver burden for patients with Parkinson’s and any usual care setting (e.g. home, care home, hospital, community, etc.) were included.
3. Study type: Observational studies to identify the relevance of caregivers burden through statistical modeling. Studies published in English or Chinese language were considered in this review.
4. Outcome measures: Caregivers burden as primary or secondary outcome and measured using validated or non-validated instruments.

#### 2.2.2. Exclusion criteria

1. Studies with mixed samples (including PwP’s caregivers and caregivers of other disease groups) were excluded unless subsample analyses were conducted on the participants of interest.
2. Studies that included caregiver burden as an independent variable rather than an outcome.
3. Studies that considered only correlates were excluded.

### 2.3. Literature search strategy

Electronic databases including PubMed, Embase, CINAHL, Web of Science, PsycINFO, Chinese Biomedical Literature Database (CBM), Chinese National Knowledge Infrastructure (CNKI), Wangfang database, and VIP database were searched using a combination of MeSH titles and keywords, such as “Parkinson’s disease,” “caregiver,” and “caregiver burden.” The search period extended from the inception of the databases to May 10, 2024. “Journal of Parkinson’s Disease,” “Parkinsonism & related disorders,” and “Movement Disorders” were also searched manually, and references were listed for studies that met the eligibility criteria. The reference list was searched without date restrictions. The search strategy for PubMed is shown in Table S1, Supplemental Digital Content, http://links.lww.com/MD/O282.

### 2.4. Study selection and data extraction

#### 2.4.1. Study selection

Literature screening was conducted in two stages. The first stage was a preliminary literature screening, in which two authors (M.N. and J.Y.) excluded literature that did not meet the inclusion criteria by reading the title and abstract, respectively, and conducted a full-text search of the eligible literature after the preliminary screening. The second stage was full-text screening, in which two authors (M.N. and J.Y.) independently performed full-text screening, and literature with different opinions was resolved by discussion or discussion with a third author (J.W.).

#### 2.4.2. Data extraction

The 2 members (M.N. and J.Y.) independently extracted information from the included literature according to the data extraction form, and the main contents of the extracted data included: (i) basic information about the literature, including title, authors, and time of publication; (ii) basic characteristics of the study population, including age, gender, race, and relationship with patients; (iii) study design, including burden of care measures, number of influencing factors included, and analysis methods used; and (iv) main results and conclusions.

### 2.5. Quality assessment of studies

We chose the checklist developed by Lixia Ge^[21]^ and colleagues to assess the quality of the included studies. The checklist is a modified checklist which Lixia Ge et al adapted from Duckitt et al^[22]^ and Zhao et al,^[23]^ and it contains five items assessed by nine separate criteria (Table S2, Supplemental Digital Content, http://links.lww.com/MD/O282). Disagreements in quality assessment were resolved through discussion. If the study does not meet the criteria, the score is zero. The quality score is calculated by the sum of all individual scores. According to the criteria, research can be scored up to 9 points. Studies included were further classified as high, medium, or low quality based on methodological quality scores (Table S3, Supplemental Digital Content, http://links.lww.com/MD/O282).

### 2.6. Data synthesis

This study used a qualitative analysis method to systematically summarize and describe the information and results of the included literature. The main contents include: (i) the basic information of the literature, including the time of publication, study population, study method, and survey instrument; (ii) the classification and classification of different influencing factors and the description of relevant results.

## 3. Results

### 3.1. Study selection results

**Figure 1.**
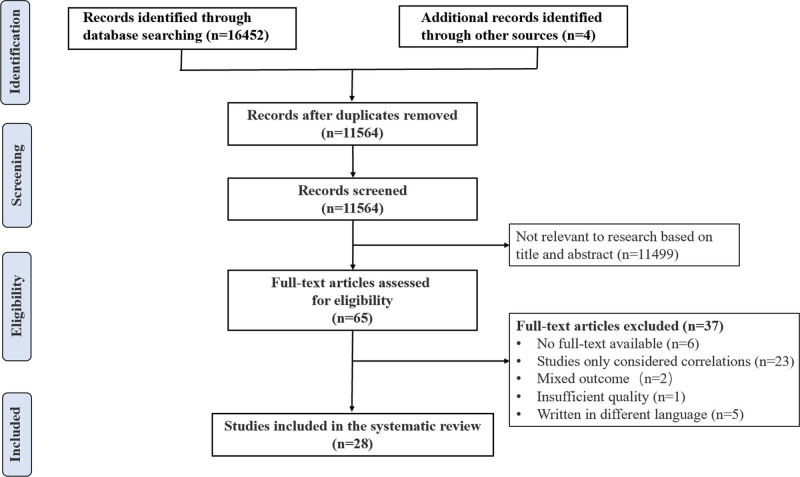
PRISMA flow diagram.

The search on 9 databases yielded 16,456 papers. After the duplicates had been removed, and the titles and abstracts had been screened, 65 papers were eligible for full text screening. After the full text screening and quality appraisal, 37 of the 65 papers were excluded and the 28 papers were included in this review. A flow diagram of the study selection is presented in Figure 1.

### 3.2. Basic characteristics of included studies

Most of the caregivers were females and most of them were spouses of PwP (Table 1). The PwP’s mean age ranged from 57.62 to 75.2 years, and the mean disease duration ranged from 5.7 to 16.38 years. Twenty-one studies reported that the mean Hoehn and Yahr stage ranged from 1.5 to 3.5 (Table 2). Of note, data were contributed in 12 different countries, increasing the representativeness and applicability of the review’s findings. Studies were conducted in the Germany (N = 3), Brazil (N = 2), Italy (N = 2), South Korea (N = 3), the USA (N = 6), the UK (N = 2), Japan (N = 1), Spain (N = 3), Sweden (N = 1), Australia (N = 1), China (N = 3), and India (N = 1).

**Table 1:** Characteristics of included studies

Study	Country	Design	Sample size	Patients’ mean age (years)	Female (%)	Relationship to patient	Caregiver burden measure	No. of variables tested	Statistical Analysis Used
Alessandro et al	Italy	Cross-sectional	N = 126	57.94 ± 12.9	88 (69.8)	Spouses (60.3%) Sons/daughters (31.7%); Brother/sister (3.2%) Other relatives (4.8%)	ZBI	5	Multiple regression analysis
Francisco et al	Brazil	Cross-sectional	N = 50	55.7 ± 13.1	44 (88.0)	Spouse (78%) Daughter/son (14%) Other family members/friends (8%)	ZCBI	20	Multiple regression analysis
Geum-Bong et al	South Korea	Cross-sectional	N = 96	Spouse (65.89 ± 10.72) offspring (44.26 ± 11.47)	50 (52.1)	Spouses (61.5%) Offspring (38.5%)	CBI	12	Multiple linear regression analysis
Haruko et al	Japan	Cross-sectional	N = 178	Maryland (63.7 ± 9.7) Yamagata (68.9 ± 9.4)	No mention	Spouses	CSI	Maryland:13; Yamagata:11	Multiple regression analysis
He Haiyan et al	China	Cross-sectional	N = 115	63.82 ± 11.64	77 (67.0)	Spouse (84.3%) Son or daughter (12.2%) Other (3.5%)	CBI	6	Multiple linear regression analysis
Hyeeun et al	South Korea	Cross-sectional	N = 42	60.0 ± 13.8	26 (61.9)	No mention	ZBI	5	Multiple linear regression analysis
Hyeeun et al	South Korea	Cross-sectional	N = 91	Spouse (66.4 ± 8.9) offspring (45.8 ± 10.9)	Spouse:25 (50.0); offspring:22 (53.7)	Spouses (54.9%) Offspring (45.1%)	ZBI	7	Multiple linear regression analysis
Iracema et al	UK	Cross-sectional	N = 71	62.72 ± 10.87	28 (39.4)	Spouses (54%) Adult children (46%)	ZBI	3	Multiple linear regression analysis
Jaya et al	India	Cross-sectional	N = 150	50.38 ± 16.04	118 (78.7)	Wife (40.67%) Husband (7.33%) Sons (12%) Siblings (6%) Daughter-in-law (20.66%) Daughter (13.33%)	CBS and BAS	6	Multiple linear regression analysis
Julie etal	USA	Cross-sectional	N = 219	64.0 ± 9.8	155 (70.7)	Spouses	FCI	3	Multiple regression analysis
L M et al	USA	Cross-sectional	N = 450	65.87 ± 10.39	333 (74.0)	Spouses (84.9%) Parent (10.2%) Sibling (1.8%) Other (5.66%)	CBI	6	Multiple linear regression analysis
Li Yunhan et al	China	Cross-sectional	N = 168	53.51 ± 11.14	152 (90.5)	Spouse (46.4%) Son or daughter (44.6%) Other (9%)	ZBI	6	Multiple linear regression analysis
M Klietz et al	Germany	Cross-sectional	N = 78	64.8 ± 11.0	42 (53.8)	No mention	PDCB	16	Multiple regression analysis
M Klietz et al	Germany	Cross-sectional	N = 118	65.4 ± 11.0	78 (66.1)	Spouse (93.2%) Child (6.8%)	PDCB	7	Multiple regression analysis
M Klietz et al	Germany	Cross-sectional	N = 119	65.4 ± 11.0	78 (65.5)	Spouse (93.2%) Child (6.8%)	PDCB	5	Multiple regression analysis
Margaret et al	USA	Cross-sectional	N = 45	70	31 (68.9)	Spouses	CBI	2	Multiple regression analysis
Maria et al	Italy	Cross-sectional	N = 51	69.0 ± 10.0	34 (66.7)	Spouse (89%) Sons (5%) Siblings (4%) Not conjunct (4%)	CBI	7	Multiple linear regression analysis
Marianne et al	Sweden	Cross-sectional	N = 65	67.0	24 (36.9)	Spouses (89.2%) Daughters (5%) Sons (3%) Brother (1.4%) Niece (1.4%)	CBS	5	Multiple regression analysis
Michael et al	Australia	Cross-sectional	N = 50	64.7 ± 10.8	34 (68)	Spouse (88%) Child (10%) Sibling (2%)	PDCB	12	Multiple linear regression analysis
Michele et al	UK	Cross-sectional	N = 704	67.05 ± 10.4	506 (71.9)	Spouse/partner (88.9%) Child (5.8%) Other family members (2.6%) Friend or other (2.6%)	CSI	8	Multiple linear regression analysis
Nancy et al	USA	Cross-sectional	N = 41	66.8	28 (68)	Spouses	ZBI	4	Multiple regression analysis
O Oguh et al	USA	Cross-sectional	N = 2476	No mention	No mention	Spouse/partner (91%) Other relatives (9.4%) Other nonpaid caregivers (0.5%)	MCSI	6	Multiple logistic regression analysis
Pablo et al	Spain	Cross-sectional	N = 562	59.62 ± 13.97	396 (70.5)	Spouse (61.2%) Children (29.5%) Other (9.3%)	ZCBI	5	Multiple linear regression analysis
Pablo et al	Spain	Cross-sectional	N = 79	61.3 ± 13.2	62 (77.5)	Spouse (76.25%) Children (18.75%) Other (3.75%)	ZCBI	5	Multiple regression analysis
Paulo et al	Brazil	Cross-sectional	N = 21	53.0 ± 12.44	17 (80)	Partners (47%) Son or daughter (38%) Grandchild (5%) Parent (5%) Daughter-in-law (5%)	ZBI	11	Multiple linear regression analysis
Santos-Garcia et al	Spain	Cross-sectional	N = 121	60.2 ± 15.0	87 (71.9)	Spouse (66.9%) Son or daughter (30.6%) other (2.5%)	ZCBI and CSI	7	Multiple linear regression analysis
Wu Ting et al	China	Cross-sectional	N = 161	Not clear	96 (59.6)	Spouse (52.2%) Son (18.6%) Daughter (19.9%) Relative (9.3%)	ZBI	8	Multiple logistic regression analysis
Zachary et al	USA	Cross-sectional	N = 175	66.1 ± 11.1	128 (73.1)	No mention	ZBI	6	Multiple regression analysis

BAS = Burden Assessment Schedule, CBI = the Caregiver Burden Inventory, CBS = Caregiver’s Burden Scale, CSI = the Caregiver Strain Index, FCI = the Family Care Inventory, MCSI = The multidimensional caregiver strain index, PDCB = Parkinson’s disease caregiver burden questionnaire, ZBI = the Zarit Burden Inventory, ZCBI = the Zarit caregiver burden interview.

**Table 2:** Summary of the key characteristics of the included studies

World region	South America: 2, North American: 6, Europe: 11, Asia: 8, Australia: 1
Ethnicity of carers (n = 5)	>87.8%white
Mean age of PwP (n = 27)	Range: 57.62–75.2years
Mean disease duration for PwP (n = 22)	Range: 5.7–16.38years
Mean Hoehn and Yahr stage for PwP (n = 21)	Range: 1.5–3.5

### 3.3. Quality of studies

Of the 28 studies included, 14 (50%) had a quality score of 7 and were therefore rated as high quality, and 8 (29%) had a quality score of 6 and were rated as moderate quality, although 6 of the studies had a quality score of 5 but were also moderate quality (Table 3).

**Table 3:** Quality assessment results of the included studies

Study	Population	Subject Selection	Study Design	Assessment of outcome	Data analysis and presentation	Total score	Quality level of studies
Alessandro et al	1	1	1	1	3	7	High
Francisco et al	1	1	1	1	3	7	High
Geum-Bong et al	1	0	1	1	3	6	Moderate
Haruko et al	1	1	1	1	3	7	High
He Haiyan et al	1	1	1	1	3	7	High
Hyeeun et al	0	1	1	1	3	6	Moderate
Hyeeun et al	1	0	1	1	3	6	Moderate
Iracema et al	1	1	1	1	3	7	High
Jaya et al	0	1	1	1	2	5	Moderate
Julie H et al	1	0	1	1	3	6	Moderate
L M et al	0	0	1	1	3	5	Moderate
Li Yunhan et al	1	1	1	1	3	7	High
M Klietz et al	1	1	1	1	3	7	High
M Klietz et al	0	1	1	1	3	6	Moderate
M Klietz et al	1	1	1	1	3	7	High
Margaret et al	0	0	1	1	3	5	Moderate
Maria et al	1	1	1	1	3	7	High
Marianne et al	0	1	1	1	2	5	Moderate
Michael et al	1	1	1	1	3	7	High
Michele et al	0	1	1	1	3	6	Moderate
Nancy et al	0	0	1	1	3	5	Moderate
O. Oguh et al	0	1	1	1	3	6	Moderate
Pablo et al	1	1	1	1	3	7	High
Pablo et al	1	1	1	1	3	7	High
Paulo et al	0	0	1	1	3	5	Moderate
Santos-Garcia et al	1	1	1	1	3	7	High
Wu Ting et al	1	1	1	1	3	7	High
Zachary et al	1	0	1	1	3	6	Moderate

### 3.4. Prevalence and severity of caregiver burden

In the included studies, caregiver burden was measured by Caregiver Burden Inventory,^[5,19,24–27]^ the Zarit Caregiver Burden Inventor,^[8,14,17,18,28–36]^ Caregiver Strain Index,^[29,37,38]^ Parkinson’s Disease Caregiver Burden Questionnaire,^[16,39–41]^ Multidimensional Caregiver Strain Inventory,^[42]^ and Family Care Inventory.^[43]^ The prevalence of caregiver burden was reported in 25 articles.^[5,8,14–19,25–33,35–42]^ Based on four papers,^[15,29,32,42]^ the prevalence of informal caregivers who typically reported mild to moderate caregiver burden ranged from 53% to 90.9%, while the prevalence of informal caregivers who reported high burden ranged from 0% to 11.2%.

### 3.5. Associated factors of caregiver burden

The associated factors of caregiver burden in Parkinson’s disease were divided into demographic factors, disease-related factors and caregiver-related factors (Table 4), which are presented in detail as follows.

**Table 4:** Summary of factors associated with higher caregiver burden

Variables	Level of Evidence	No. of caregivers in high-quality study	No. of caregivers in moderate-quality study
Patient characteristics			
Demographics			
Gender	Conflicting		
Male			2476^[42]^
Female			142^[5]^
More severe degree of PD	Moderate	80^[36]^ + 161^[33]^	2476^[42]^ + 65^[24]^ + 45^[25]^ + 150^[34]^
Longer disease duration	Moderate	575^[8]^ + 168^[14]^	
Lower quality of life	Strong	124^[16]^ + 126^[15]^ + 115^[27]^	2476^[42]^ + 175^[35]^
QoL-limitations in physical mobility	Limited		704^[38]^
Motor symptoms			
Higher scores of UPDRS-Motor	Moderate	124^[16]^	41^[30]^ + 150^[34]^
Decreased verbal fluency	Limited		2476^[42]^
Falls	Limited	62^[37]^	
Lower ADL/UPDRS-ADL scores	Strong	121^[29]^ + 51^[26]^ + 115^[27]^ + 119^[41]^	41^[31]^ + 219^[43]^ + 118^[40]^
Disability	Moderate	575^[8]^ + 80^[36]^	
Sleep disorders	Limited	50^[17]^	
Neuropsychiatric symptoms			
Psychosis	Moderate	50^[17]^ + 575^[8]^	
Higher scores of UPDRS-Mentation	Moderate		42^[18]^ + 50^[30]^
Impaired attention	Limited	22^[28]^	
Depression	Strong	21^[28]^ + 54^[37]^ + 121^[29]^ + 50^[39]^ + 161^[33]^	219^[43]^
Anxiety	Limited		450^[19]^
Hallucinations	Limited		450^[19]^
Agitation	Limited		450^[19]^
Nighttime behaviors severity	Limited		450^[19]^
Apathy	Moderate	575^[8]^	450^[19]^
Deficits in visuoconstruction	Limited	50^[39]^	
Delayed recall	Limited		219^[43]^
Perceived worse control over symptoms	Limited		45^[25]^
Medications			
Presence of concomitant medications	Limited		2476^[42]^
Higher level of levodopa equivalent daily dose	Limited	21^[28]^	
Caregiver characteristics			
Demographics			
Elder age	Limited	115^[27]^	
Lower level of education	Limited	168^[14]^	
Lower quality of life	Moderate	126^[15]^ + 79^[36]^	175^[35]^ + 118^[40]^
Depression	Strong	50^[17]^ + 116^[37]^ + 50^[39]^ + 79^[36]^	42^[18]^ + 50^[30]^ + 65^[24]^ + 175^[35]^
Anxiety	Moderate	79^[36]^	175^[35]^
Caregiving situation			
Longer time of caregiving	Moderate	50^[17]^	59^[5]^ + 150^[34]^
Caregiver’s change in work	Limited	126^[15]^	
Negative coping	Moderate	168^[14]^	65^[24]^
Less understanding of the disease	Limited		59^[5]^
Lower self-efficacy	Limited	161^[33]^	
Different study site	Limited		175^[35]^
Supporting resources			
Lack of social support	Moderate	54^[37]^ + 161^[33]^	2476^[42]^ + 704^[38]^ + 41^[30]^ + 41^[31]^
Self-paying medical care	Limited	168^[14]^	

ADL = activities of daily living, UPDRS = Unified Parkinson’s Disease Rating scale.

#### 3.5.1. Demographic factors

##### 3.5.1.1. Patient characteristics

Eleven of the 28 studies in the literature examined the relationship between patient gender and caregiver burden among 4171 participants.^[5,15,17,25–27,37–40,42]^ Two articles showed that gender was a predictor of caregiver burden and that caregiver burden was higher when the patient was male^[42]^; in the other article,^[5]^ the opposite was found. Other studies with 1553 participants did not reach the same conclusion.

##### 3.5.1.2. Caregiver characteristics

In 10 articles with 1227 participants,^[5,15,26,27,33–37,39]^ the age of caregivers of Parkinson’s patients was examined as an influencing factor, with only 1 study^[27]^ suggesting that the older the caregiver, the greater the caregiver burden. In addition, seven articles examined the relationship between caregiver education and caregiver burden,^[5,14,26,27,29,33,37]^ and only one article with 168 participants showed a significant association between caregiver education and caregiver burden.^[14]^

#### 3.5.2. Disease-related factors

##### 3.5.2.1. Severity of Parkinson’s disease

Fourteen studies with a total of 3801 participants discussed the relationship between severity of Parkinson’s disease(used the Hoehn & Yahr stage) and caregiver burden,^[5,15,17,18,24,25,27,29,33,34,36,37,39,42]^ and 6 of them suggested that the higher H&Y stage, the increased caregiver burden.^[24,25,33,34,36,42,44]^

##### 3.5.2.2. The duration of Parkinson’s disease

The relationship between the duration of Parkinson’s disease and caregiver burden was discussed in 15 studies with 4955 participants,^[5,8,14,24,26,27,29,32,34,36,38–42]^ 2 of these studies reported that the longer the disease duration, the higher caregiver burden score.^[8,14]^ Nevertheless, 13 studies did not find this factor to be a significant contributor.

##### 3.5.2.3. Quality of life

Eight studies with 3957 participants reported a positive association between impaired quality of life (QoL) and caregiver burden as measured by the Parkinson’s Disease Questionnaire (PDQ),^[15,16,27,35,38,40–42]^ with one study specifically referring to the Mobility dimension of the PDQ, suggesting that limitations in physical activity were strongly associated with increased caregiver burden.^[38]^ However, the other six studies did not support this conclusion.^[24,29,34,36,40,41]^

##### 3.5.2.4. Higher scores of UPDRS-Motor

Motor symptoms as measured by the Unified Parkinson’s Disease Rating Scale (UPDRS) were examined as a factor in 13 studies.^[16,18,26–30,32–35,37,39]^ Three of these studies,^[16,30,34]^ which included 365 participants, showed that the severity of motor symptoms in Parkinson’s patients was significantly associated with caregiver burden, while the other 10 articles did not reach this conclusion.

##### 3.5.2.5. Reduced verbal fluency and falls

In 28 articles, two of these discussed the relationship between falls and caregiver burden,^[37,42]^ one study, with 178 participations, reported falls was a significant contributor.^[37]^ In addition, only one study^[42]^ showed that reduced verbal fluency was significantly associated with increased caregiver burden.

##### 3.5.2.6. Lower ADL/UPDRS-ADL scores

Four high-quality articles,^[26,27,29,41]^ and three moderate-quality article^[31,40,43]^ all examined the relationship between the ability to perform activities of daily living (ADL) and caregiver burden in Parkinson’s patients, and all showed that ability to perform ADLs was one of the predictors of caregiver burden, i.e., the lower the patient’s ability to perform ADL, the higher the caregiver burden.

##### 3.5.2.7. Disability

Of the four studies that included 883 participants,^[8,17,36,37]^ two suggested that disability in Parkinson’s patients was significantly associated with increased caregiver burden.^[8,36]^ Disability was measured by the Clinical Impression of Severity Index for PD (CISI-PD) and the SCOPA-Motor scale (SMS-ADL), respectively, i.e., the higher scores of scales, the higher burden of caregiver.

##### 3.5.2.8. Sleep disorders

Only one high-quality article reported that sleep disorders were an influential factor in caregiver burden.^[17]^ Sleep was measured by the Scales for Outcomes in Parkinson’s Disease-Sleep scale (SCOPA-Sleep), i.e., higher scores reflected more severe sleep problems. Other 27 studies did not mention that.

##### 3.5.2.9. Psychosis

Two studies measured the relationship between psychiatric symptoms and caregiver burden in Parkinson’s patients using the Scale for Evaluation of Neuropsychiatric Disorders in PD (SEND-PD) and the Parkinson’s psychosis rating scale (PPRS),^[8,17]^ and both of these studies, which included 625 participants, reported that the degree of psychiatric symptoms suffered by patients were proportional to caregiver burden.

##### 3.5.2.10. Higher scores of UPDRS-Mentation

Seven articles investigated the relationship between mentation and caregiver burden as measured by the Unified Parkinson’s Disease Rating Scale (UPDRS) in 667 participants,^[16,18,30,32,33,37,39]^ with two studies suggesting that higher patient scores on the UPDRS-Mentation were strongly associated with higher caregiver burden.^[18,30]^

##### 3.5.2.11. Depression

The correlation between depression and caregiver burden in Parkinson’s patients was explored in 11 articles that included 1449 participants,^[16,17,19,28,29,33,36,37,39,41,43]^ with 6 articles reporting a significant correlation between patient depression and caregiver burden, i.e., higher levels of patient depression were associated with higher caregiver burden.^[28,29,33,37,39,43]^

##### 3.5.2.12. Anxiety

Four articles investigated the relationship between anxiety levels and caregiver burden in 706 participants,^[15,19,36,37]^ and only 1 study suggested a significant association between anxiety symptoms and increased caregiver burden in Parkinson’s patients.^[19]^

##### 3.5.2.13. Hallucinations, agitation and nighttime behaviors severity

One moderate-quality article with 450 participants also reported that hallucinations, agitation, and nocturnal behavior, as all measured by Neuropsychiatric Inventory Questionnaire (NPI-Q), were significantly associated with caregiver burden in Parkinson’s patients.^[19]^ Other 27 studies did not discuss that relationship.

##### 3.5.2.14. Apathy

In 2 studies with 1025 participants, both suggested a significant association between apathy symptoms and increased caregiver burden in Parkinson’s patients.^[8,19]^

##### 3.5.2.15. Deficits in visuoconstruction

The relationship between patients’ cognitive function and caregiver burden was explored in 12 studies that included 4251 participants,^[8,15–17,27,28,33–35,37,39,42]^ and only 1 suggested that impairment of visuoconstruction in cognitive function was significantly related to caregiver burden.^[39]^

##### 3.5.2.16. Delayed recall

One moderate-quality study explored the association between delayed recall in Parkinson’s patients and caregiver burden by using the Selective Reminding Test.^[43]^ When PD patients exhibited worse delayed recall, their caregivers reported a greater burden. Other 27 studies did not mention that.

##### 3.5.2.17. Medication factors

The association between medication combinations and caregiver burden in Parkinson’s patients was tested in 3 articles,^[28,37,42]^ and the presence of combination medications such as antidepressants or anxiolytics and higher daily doses of levodopa were associated with caregiver burden.^[28,42]^

#### 3.5.3. Caregiver related factors

##### 3.5.3.1. Quality of life

A total of 5 studies examined the relationship between caregiver QoL and caregiver burden,^[15,17,35,36,40]^ three studies with 380 participants suggested that QoL was significantly associated with caregiver burden, with lower caregiver quality of life associated with higher caregiver burden.^[15,35,36]^

##### 3.5.3.2. Depression and anxiety

Nine studies investigated the relationship between caregiver depression and caregiver burden,^[16–18,24,30,35–37,39]^ with eight studies (including 4 high quality and 4 medium quality) with 627 participants indicating that the higher the level of caregiver depression, the higher the caregiver burden.^[17,18,24,30,35–37,39]^ In addition, 2 studies with 354 participants both reported a positive association between caregiver anxiety and caregiver burden.^[35,36]^ Nevertheless, other 2 studies didn’t reach the same conclusion.^[17,39]^

##### 3.5.3.3. Caregiving duration

Caregiving duration was explored as a correlate of caregiver burden in 13 articles,^[5,15–17,19,27,32–34,36,38,39,42]^ including 3 articles with 259 participants that identified caregiving duration as a predictor of caregiver burden, with longer or more frequent caregiving duration associated with higher caregiver burden.^[5,17,33]^

##### 3.5.3.4. Coping style

Regarding the relationship between caregiver coping style and caregiver burden, the relationship was reported in 2 articles with a total of 233 participants,^[14,24]^ i.e., the more likely a caregiver was to successfully cope with a stressful situation or to cope actively,^[14,24]^ the lower the caregiver burden, i.e., Coping style was measured by the sense of coherence (SOC) scale, the stronger the SOC, the more likely it was that the person would be able to cope successfully.^[24]^ However, other 26 articles did not draw this conclusion.

##### 3.5.3.5. Social support

A total of 7 studies including 3498 participants investigated the correlation between social support and caregiver burden,^[30–33,37,38,42]^ with 6 of them all concluding that more social support reduces caregiver burden.^[30,31,33,37,38,42]^

## 4. Discussion

This is a systematic review summarizing factors associated with the higher caregiver burden of informal caregivers for PwP in different settings around the world. Our review found that lower quality of life of PwP and lower ADL scores of PwP (strong evidence) increased the care burden of informal caregivers. The reduced quality of life of patients represents the greater impact of the disease on patients, while the decrease in ADL levels also suggests that patients are less able to take care of themselves and become more dependent on caregivers for feeding, dressing, and washing, both of which add more caregiving tasks and mental burdens to caregivers. Therefore, health care professionals should encourage PwP to actively engage in rehabilitation exercises to improve their self-care ability and improve their quality of life, thereby reducing the caregiver burden.

This review revealed that PwP’s depression is an indicator (strong evidence) of informal caregiver burden. Previous studies have revealed that depression is one of the important non-motor features in Parkinson’s disease, which occurs at high frequency and influences many other clinical aspects of the disease.^[45,46]^ A systematic review indicated that the pooled prevalence of major depression was 22.9%.^[47]^ Several longitudinal analyses suggest that depression and its treatment influence the course of motor symptoms, which decreased the quality of life of PwP, and as a result, causing a huge burden on caregivers.^[48,49]^ A systematic review on managing depression in individuals with Parkinson’s disease indicates that selective serotonin reuptake inhibitors (SSRIs) and cognitive-behavioral therapy (CBT) are currently regarded as first-line treatment options.^[50]^ Despite these options, the response to treatment varies widely, and tailoring interventions to each patient’s unique needs remains essential. Thus, healthcare professionals should provide PwP with adequate depression treatment.^[51–53]^ Timely medical help and medication support can improve patients’ symptoms and thus reduce caregivers’ burden of care.^[54]^

Depression in informal caregivers is a risk factor of care burden. Depression is characterized by persistent low mood and negative cognitive appraisals towards oneself that can complicate coping with external pressure, and is often accompanied by symptoms such as decreased sleep quality and loss of appetite. Also, depression in informal caregivers and caregiving burden may be an interactive process. Therefore, informal caregivers’ depression should be treated. Studies have shown that giving caregivers adequate emotional and social support is effective in reducing caregiver anxiety and depression levels.^[55,56]^ Furthermore, interventions such as early palliative care, educational resources, and psychotherapy for families of individuals with Parkinson’s disease have demonstrated efficacy in alleviating caregiver anxiety and enhancing overall wellbeing.^[4,57]^ Expanding research to better understand the nuanced experiences of caregivers may also inform targeted interventions that address the unique challenges of Parkinson’s caregiving, ultimately contributing to a more sustainable and holistic care environment for both patients and their families.

This review also identified 12 moderate evidence factors associated with the higher caregiver burden. Both the severity of the disease (moderate evidence) and the duration of the disease (moderate evidence) place an increased burden on caregivers. At the late-stage of PwP, an increasing number of complex symptoms, both motor (i.e. walking difficulties in daily life) and non-motor symptoms (i.e. fatigue),^[58]^ commonly generate PwP experiencing a progressive loss of functional independence, requiring increasing levels of assistance of a caregiver over time. While the physical function of both the patient and the caregiver declines, the caregiver’s ability to cope with the burden decreases. Therefore, medical staff should guide PwP to actively engage in early rehabilitation exercises such as Tai Chi and other physical exercises to avoid the occurrence of disabling conditions and prevent the disease from progressing too quickly,^[59,60]^ thereby reducing the burden on caregivers.

Psychosis, UPDRS-Mentation and apathy are all manifestations of neuropsychiatric symptoms. Neuropsychiatric symptoms in PwP are in significant correlation with caregiver burden.^[61]^ PwP who suffer from neuropsychiatric symptoms are prone to a variety of caregiving burdens for caregivers, such as: difficulty in caregiving, financial burden, and emotional stress.^[62]^ Thus, there is a need to raise awareness of PwP among medical staff and adopt more effective and safer drug treatments to improve the quality of care for PwP and reduce the huge burden on their caregivers.^[63]^

Moderate evidence suggests that some characteristics of caregivers also influence caregiving burden, such as: lower quality of life, anxiety, longer time of caregiving and negative coping. When caregivers perceive their quality of life to be poorer, they will feel that their caregiving burden is heavier. Conversely, previous research has also indicated that when caregivers’ caregiving burden is higher, their perceived quality of life is also worse.^[64]^ In conclusion, caregiver quality of life and caregiving burden may be an interactive process, and future research could design a longitudinal study to explore changes in the relationship between the two. However, for medical professionals, changes in caregiver quality of life will inevitably improve the caregiving burden of patients. The results of this review suggest that caregiver anxiety is an influential factor in caregiving burden. This may be related to the caregiver’s lack of hope for the future, concern about the large financial drain, and fear of not being able to care for the patient to affect recovery. Some strategies need to be adopted to reduce caregivers anxiety, such as identifying social support systems and allocation of resources, providing psychological support, and so on.^[35,65]^

The amount of time spent providing daily care is considered direct care time, and direct care time is an important factor in caregiver burden. The results of this study showed that informal caregivers with longer caregiving time had a higher caregiver burden than informal caregivers who provided less time (moderate-evidence), which is consistent with the findings among caregivers of patients with dementia.^[66]^ Medical professionals should fully explore ways to reduce the length of care time for Parkinson’s disease caregivers, for example, they can try to implement day services, and if it is not possible to reduce the length of care for caregivers, multidisciplinary care team can provide remote care guidance for PwP caregivers, which can effectively improve the caregiving ability of caregivers and reduce their psychological burden.^[67]^ Additionally, home care increasingly becomes incapacitating at advanced Parkinson’s disease stage, there may be a transition into institutional care. According to several research, the caregiver’s burden decreases after the patients’ transition to institutional care due to the shorter length of care.^[68,69]^ However, some informal caregivers are less likely to institutionalize the individual they provide care for. On the one hand, feelings of guilt, fear of financial needs, and other reasons may affect the process of decision-making concerning the transition of PwP into institutional care^[68]^; on the other hand, due to some cultural norms, such as the concepts of *marianismo*(self-sacrifice) and *familismo* (familism) in Latin America may encourage caregiving of PwP among women and other family members,^[70,71]^ i.e., higher levels of *familismo* were associated with lower caregivers burden.^[72]^ Family members may view the opportunity to care for their loved one as meaningful and gratifying instead of burdensome. Therefore, future studies should take into account how cultural norms and values affect caregiver burden.

Caring for PwP usually brings pressure to informal caregivers. Caregivers will have discomfort and the degree of discomfort due to the stimulation of stressors, which is mainly affected by caregivers’ coping styles. When caregivers adopt negative coping style, caregivers can not eliminate the pressure, they will feel the heavy burden of care. Medical staff should evaluate the coping style of caregivers and take intervention measures (e.g., group spiritual counseling for caregivers,^[35]^ cognitive behavioral therapy^[73]^) to promote the positive coping of caregivers. The results of this review also showed that social support (moderate-evidence) is associated with the informal caregivers’ burden. The lower the level of social support, the higher the caregiver burden. Zongfang Yang et al^[56]^ have pointed out that caregiver social support is a chain mediating variable between patients’ motor dysfunction and caregiver burden, indicating that improving caregivers’ social support is one of the ways to reduce caregiver burden. Comprehensive patient symptom management, support groups and psychoeducational interventions have the potential to improve caregiver’s social support.^[74]^

This study also identified a conflicting factor: gender. Future research needs to further explore the impact of gender on caregiver burden. And there are some other factors of low-evidence also identified. Some of the factors related to PwP’s characteristics; some related to informal caregivers’ characteristics; some related to caregiving situation; while others related to supporting resources. In the future, rigorous research should be further designed to improve the level of evidence of these factors.

The main sources of caregiver burden among informal PwP caregivers were extensively reviewed in the current study. The findings of this systematic review showed that the main causes of caregiver burden among informal caregivers were motor issues (i.e., ADL), neuropsychiatric symptoms, and caregiver psychological components of PwP. Future studies should explore additional potential caregiver burden factors. The majority of studies that focus on caregiver burden to date are cross-sectional, but longitudinal studies are required. Healthcare professionals can provide the proper support to relieve the caregiver burden and enhance the quality of caregiving for PwP by understanding all of the factors that affect PwP’s caregiver burden. The parallels and variations between caregivers burden in these various cultures have not yet been studied. It is important to evaluate caregiver burden with cultural differences in future studies.

## 5. Limitations

There are several limitations in this systematic review. Firstly, the evidence all comes from cross-sectional surveys, there is no longitudinal study, and further exploration is needed on how the factors have changed over time. Secondly, 29 out of 38 factors were only covered once or twice. Moreover, the different care burden measures for care burden caused a high level of heterogeneity. Thirdly, although our search strategy led us to a wide range of studies in electronic databases covering a variety of topics, and we contacted authors of included papers to identify additional journal articles, however, we did not search the gray literatures that were not published in journals included in major databases. As a result, relevant findings may have been missed. Fourthly, our search strategy was limited to English and Chinese, which may have led to the omission of studies published in other languages.

## 6. Conclusion

Factors related to higher caregiver burden among informal caregivers of PwP were summarized in this systematic review. Among the 38 identified factors, 4 were considered of strong evidence, 12 were supported by moderate evidence, 21 were supported by limited evidence, while 1 was a conflicting factor. This review suggests that health care professionals should consider modifiable factors such as quality of life, anxiety, time spent caring, coping styles, and social support when supporting informal caregivers caring for patients with Parkinson’s disease. Future research need to use a longitudinal design to elucidate factors associated with informal caregiving burden and to further explore mediators and moderators of informal caregiver burden.

## Acknowledgments

The authors sincerely thank Meng-Nan Liu for the writing guide.

## Author contributions

**Conceptualization:** Jinwen Wu, Yuan Zou.

**Formal analysis:** Mengnan Liu, Jinyi Xue.

**Investigation:** Mengnan Liu, Jinyi Xue.

**Methodology:** Jinwen Wu, Ziwen Deng.

**Resources:** Mengnan Liu, Mingtai Chen.

**Supervision:** Xue Yang.

**Writing – original draft:** Jinwen Wu, Mengnan Liu.

**Writing – review & editing:** Jinwen Wu, Shufei Zhao.

## Supplementary Material


