# Supplementary material for: Factors associated with higher caregiver burden among informal caregivers of Parkinson’s disease: A systematic review
Source: Medicine (Baltimore). 2025 Jan 24;104(4):e41275. doi: 10.1097/MD.0000000000041275 (PMC11771715; doi:10.1097/MD.0000000000041275)
Supplement: Supplementary file 1 [file medi-104-e41275-s001.docx]

Supplementary Table 1. Example search strategy for PubMed

| Database search strategy |
| --- |
| 1 parkinsonian disorders[Mesh]  2 parkinson disease[Mesh]  3 parkinson*[tiab]  4 PwP[tiab]  5 1 or 2 or 3 or 4  6 family[MeSH]  7 caregivers[MeSH]  8 carers[MeSH]  9 caregiver*[tiab]  10 carer*[tiab]  11 famil*[tiab]  12 relative*[tiab]  13 parent*[tiab]  14 spous*[tiab]  15 partner* [tiab]  16 husband[tiab]  17 wife[tiab]  18 sibling*[tiab]  19 brother[tiab]  20 sister[tiab]  21 6 or 7 or 8 or 9 or 10 or 11 or 12 or 13 or 14 or 15 or 16 or 17 or 18 or 19 or 20  22 caregiver burden[Mesh]  23 burden*[tiab]  24 strain[tiab]  25 stress[tiab]  26 distress[tiab]  27 suffer*[tiab]  28 overload*[tiab]  29 burnout[tiab]  30 exhaustion[tiab]  31 adjust*[tiab]  32 22 or 23 or 24 or 25 or 26 or 27 or 28 or 29 or 30 or 31  33 5 and 21 and 32 |

Supplementary Table 2. Criteria for Assessment of Methodological Quality of Observational Studies

| **Item** | **Criterion** | **Score** |
| --- | --- | --- |
| Population | Sample size≥50 and participation rate ≥80% | 1 |
| Subjects selection | For cohort and case-control studies: selected subjects were representative of the study population; for cross-sectional studies: inclusion criteria of primary family caregivers were clearly defined, and subjects and settings were described in detail | 1 |
| Study design | Cohort design with the duration of follow-up reported | 2 |
|  | Case-control or cross-sectional design | 1 |
|  | Withdrawals≤20% | 1 |
| Assessment of outcome | Validated burden assessment instrument was used | 1 |
| Data analysis and presentation | Appropriate statistical analyses were used | 1 |
|  | Multiple regression/multivariate analysis was performed | 1 |
|  | Frequencies of caregiver burden were reported | 1 |

Supplementary Table 3. Criteria for Assessment of Quality Level of Studies and Best-Evidence Synthesis

| **Item** | **Level** | **Subject Selection** |
| --- | --- | --- |
| Quality level of studies | High | Multivariate analysis performed and had a quality score≥7 |
|  | Moderate | Multivariate analysis performed and had a quality score<7, or no multivariate analysis performed but had a quality score>5 |
|  | Low | No multivariate analysis performed but had a quality score≤5 |
| Level of evidence | Strong | Minimum of 3 high-quality studies with generally consistent findings |
|  | Moderate | Minimum of 2 moderate-quality studies with generally consistent findings |
|  | Limited | Minimum of 1 low-quality study with generally consistent findings |
|  | Conflicting | Converse findings in >25% of the studies |
|  | None | No studies could be found |
